# IoT Workload Emulation for Data Centers

**DOI:** 10.12688/openreseurope.13079.1

**Published:** 2021-03-24

**Authors:** Thomas Batz, Reinhard Herzog, Jon Summers, Kym Watson

**Affiliations:** 1 Fraunhofer Institute of Optronics, System Technologies and Image Exploitation IOSB, Karlsruhe, Germany; 2 Research Institutes of Sweden, Swedish Institute of Computer Science (RISE SICS), North Luleå, Sweden; 3 School of Mechanical Engineering, University of Leeds, Leeds, UK

**Keywords:** Internet of Things, IoT, Benchmark, SmartCity, Predictive Maintainence

## Abstract

The Internet of Things (IoT) domain has been one of the fastest growing areas in the computer industry for the last few years. Consequently, IoT applications are becoming the dominant work load for many data centers. This has implications for the designers of data centers, as they need to meet their customers' requirements.

Since it is not easy to use real applications for the design and test of data center setups, a tool is required to emulate real applications but is easy to configure, scale and deploy in a data center. This paper will introduce a simple but generic way to model the work load of typical IoT applications, in order to have a realistic and reproducible way to emulate IT loads for data centers.

IoT application designers are in the process of harmonizing their approaches on how architectures should look, which building blocks are needed, and how they should interwork. While all IoT subdomains are diverse when it comes to the details, the architectural blueprints are becoming more and more aligned. These blueprints are called reference architectures and incorporate similar patterns for the underlying application primitives.

This paper will introduce an approach to decompose IoT applications into such application primitives, and use them to emulate a workload as it would be created by the modeled application. The paper concludes with an example application of the IoT Workload Emulation in the BodenTypeDC experiment, where new cooling approaches for data centers have been tested under realistic work load conditions.

## I. Introduction

The motivation for the development of an IoT Workload Emulation originated in the H2020 BodenTypeDC project. This project developed a highly energy efficient data center that applies new cooling concepts and is able to operate under real workload conditions at energy efficiency levels beyond the state of the art. This required a method to create a workload for the IT equipment that behaves just like a real application provided by a typical data center customer. But it must be easy to handle, reproducible, scalable and configurable to create a broad range of load levels to evaluate the data center operational efficiency. Section II gives an overview of the modeling and implementation approach of the IoT benchmark.

From the requirements of the BodenTypeDC project it was clear that the fast growing “Internet of Things (IoT)” domain shall be used as an example for typical applications. Such IoT applications are characterized by huge amounts of sensor data, high data volumes, complex event-driven processing paradigms, and demanding processing cycles.

The first challenge was to find a method for modeling such IoT application characteristics in a detailed enough way to be considered to be realistic, but at the same time easy to use and flexible enough to scale. The method shall provide a straightforward way to model different variants of IoT application examples.

The solution was to describe the desired IoT application as a collection of “User Stories”, decomposed into “Use Cases” and mapped onto “Application Primitives”. These application primitives represent elements from IoT reference architectures and are implemented as executable components of the IoT Benchmark. They are described in Section III.

## II. Benchmark implementation approach

### A. Background on IT-benchmarking

The definition of a synthetic workload is described by Shishira *et al.* ^1^ as workloads that should be representative of real workloads. They develop a very well-structured description of workload classification, in order to categorize different workload tools. According to the classification schema proposed by Shishira *et al.*, an IoT benchmark falls into one of the following categories:

- Based on generation (hybrid): The IoT benchmark is based on a combination of synthetic and real components to emulate a realistic application based load pattern.
- Based on application: The IoT benchmark is able to emulate application behavior of typical IoT applications, such as Smart City installations, or Industrial IoT solutions.

Yin *et al.* ^2^ identify two synthetic workload generation approaches, namely empirical based on sampling of application traces that are replayed and analytical, which are based on mathematical models ^3^. The IoT benchmark described in this paper is an approach to build IoT type workloads using an arrangement of computing components, which are essentially algorithmically defined and could be considered as a third approach that it is neither empirical nor mathematical.

### B. Architecture approach

In the advent of the IoT domain, many different architectural frameworks have been developed and consequently many different design patterns have been used and are still in use. Although different application fields have different features, the key patterns turned out to be very similar.

There are several reference architectures available, such as the *Reference Architecture Model Industrie 4.0* (RAMI4.0, ^4^), the *Industrial Internet Reference Architecture* (IIRA, ^5^), the *ISO/IEC – Internet of Things (loT) - Reference Architecture ^6^*, and the *Internet of Things - Architecture (IoT-A, ^7^)*.

RAMI4.0 focuses on the manufacturing sector. IIRA is very popular in diverse sectors including manufacturing, energy, mining, retail, health care, smart cities and transportation. They all share common elements, such as IoT device abstraction layers, data management services for accessing and streaming data, as well as business application layers.

**Figure 1.**
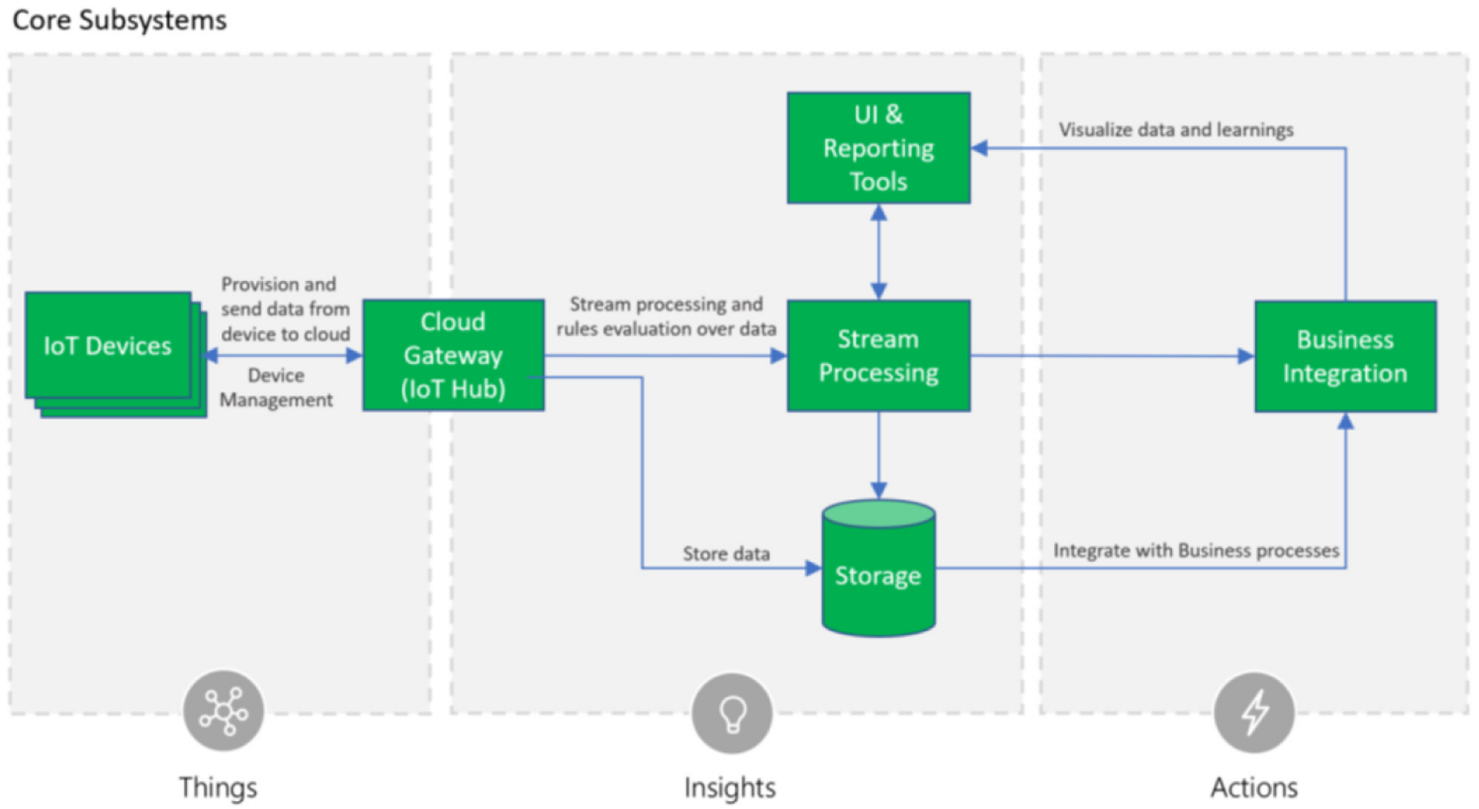
Microsoft IoT Reference Architecture ^8^.

A nice representation of these common elements is used in the Microsoft IoT reference architecture (see 8). Figure 1 shows the functional areas of the core system. This high level abstraction is a very generic abstraction of the most relevant components in an IoT application and has been chosen as a blueprint for the IoT benchmark components.

It shows the IoT devices located outside the data center, a gateway component to receive the data, a storage component for data persistence, a stream processing component for all event-based calculations and UI and reporting tools to present the acquired and processed data to the user. The component business integration represents any type of application using the IoT data, either with a query-based access pattern, or with an event-based processing pattern.

To be as close as possible to a real IoT application, the IoT benchmark populates the above functional areas of the IoT reference architecture with benchmark components to emulate their typical behavior in terms of computing and communication requirements. The benchmark components are called Application Primitives and will be explained in section II-C. They are designed to be assembled into sets of application primitives, to represent operational applications as they are used in the domains such as the industrial IoT domain, as well as in the environmental monitoring and the smart city domain.

These generic components will be instantiated according to the requirements of a specific use case. While the *Things*-Area is located outside the data center, the areas *Insights* and *Actions* are typically located within the data center.

### C. Implementation

The IoT Benchmark has been implemented as a set Java applications, where each application is implementing one specific application primitive, introduced in section II-B. The software has been made available under the open source under the GNU Lesser General Public License version 3, and can be retrieved from the GitHub repository https://github.com/FraunhoferIOSB/IoT-Benchmark ^9^. This repository includes the build definitions for the MAVEN ^10^ build tool. The build creates stand-alone Java applications and docker containers ^11^ for each application primitive.

**Figure 2.**
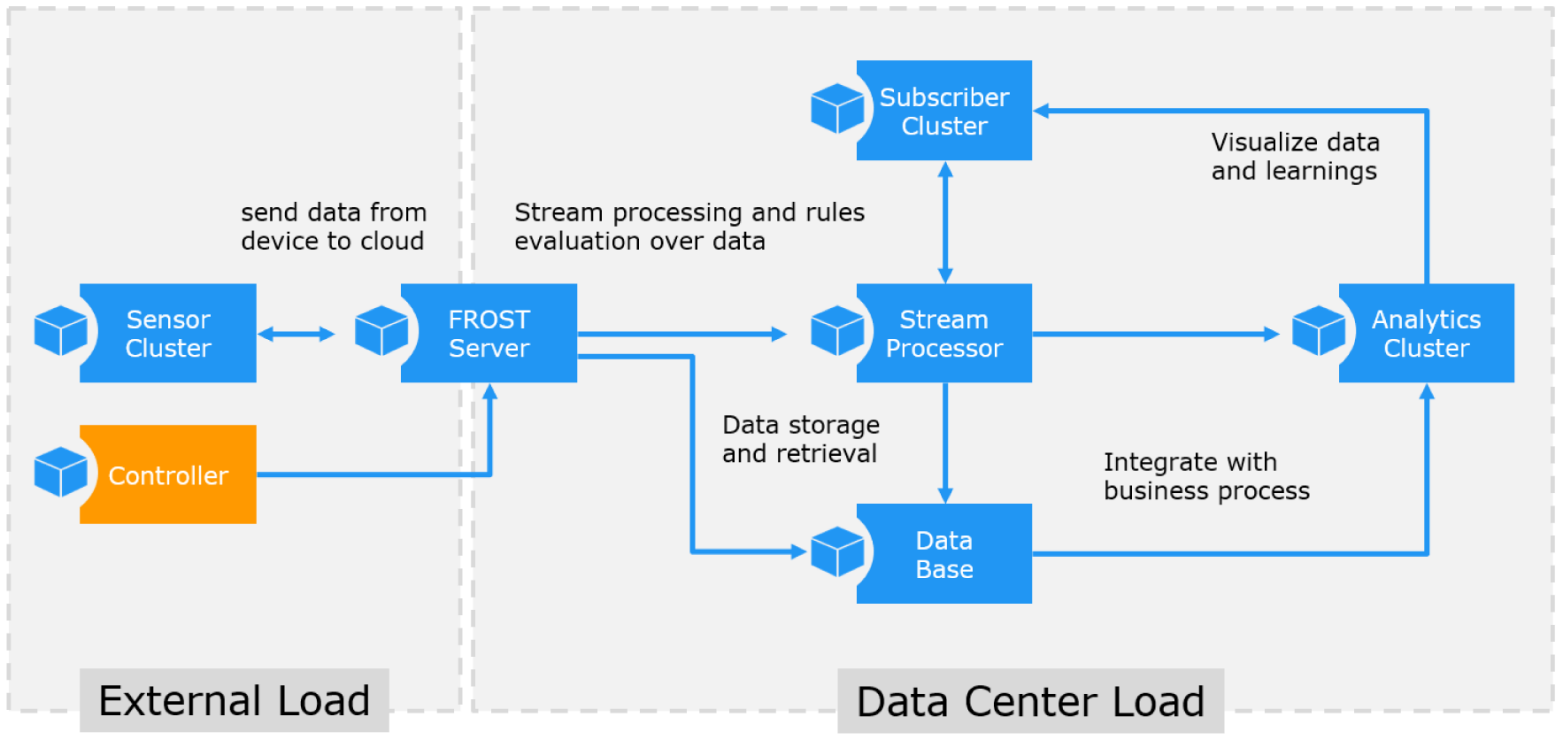
IoT Benchmark application components.

The application primitives shown in Figure 2 are implementing a behavior which is characteristic for one of the core subsystems as defined in the Microsoft IoT reference architecture. The applications can be configured to emulate specific workload patterns and multiple instances can be started to distribute the workload on several hosts. All applications are designed to cooperate as a processing cluster. The sensor cluster creates sensor observations which are subscribed by the stream processors and are accessed by the analyzer. Each application has a session parameter, which is used to link applications. Applications with the same session id use the same data sets, and applications with different session IDs are independent.

- The *FROST ^® ^Server* is a real application and is used to provide a realistic IoT data storage. It is an open source application and available for the benchmark without any license restrictions. The FROST server is used to coordinate the application primitives and to implement the data streams. It must be running before any of the other application primitives can be started. For details see https://github.com/FraunhoferIOSB/FROST-Server
- *The Sensor Cluster* application emulates a set of sensors. The sensors create observations at a given rate defined by the PERIOD parameter, which defines the delay in milliseconds before the next observations are created. The lower bound is restricted by the processing power of the host running the application.
- The *Stream Processor* application subscribes to the incoming sensor observations, and the received values are used to trigger new observations. The load behavior therefore depends on the volume and rate of the incoming data. The COVERAGE parameter defines the coverage of the subscribed data streams.
- The *Subscriber Cluster* implements an application behavior typical for data consumers. A data consumer can be located outside the data center, in order to emulate external data consumers such as data dash boards displayed in web browsers. A pure data subscriber behavior is also typical for many backend applications located within the data center.
- The *Analytics Cluster* application is used to emulate complex queries which are scheduled in a regular interval. The load behavior therefore depends on the number of jobs running and the number of analytic loops executed in each job.
- The *Controller* application implements a command line interface to the benchmark tools. It is used to start and stop experiments and does not create any load by itself.

**Figure 3.**
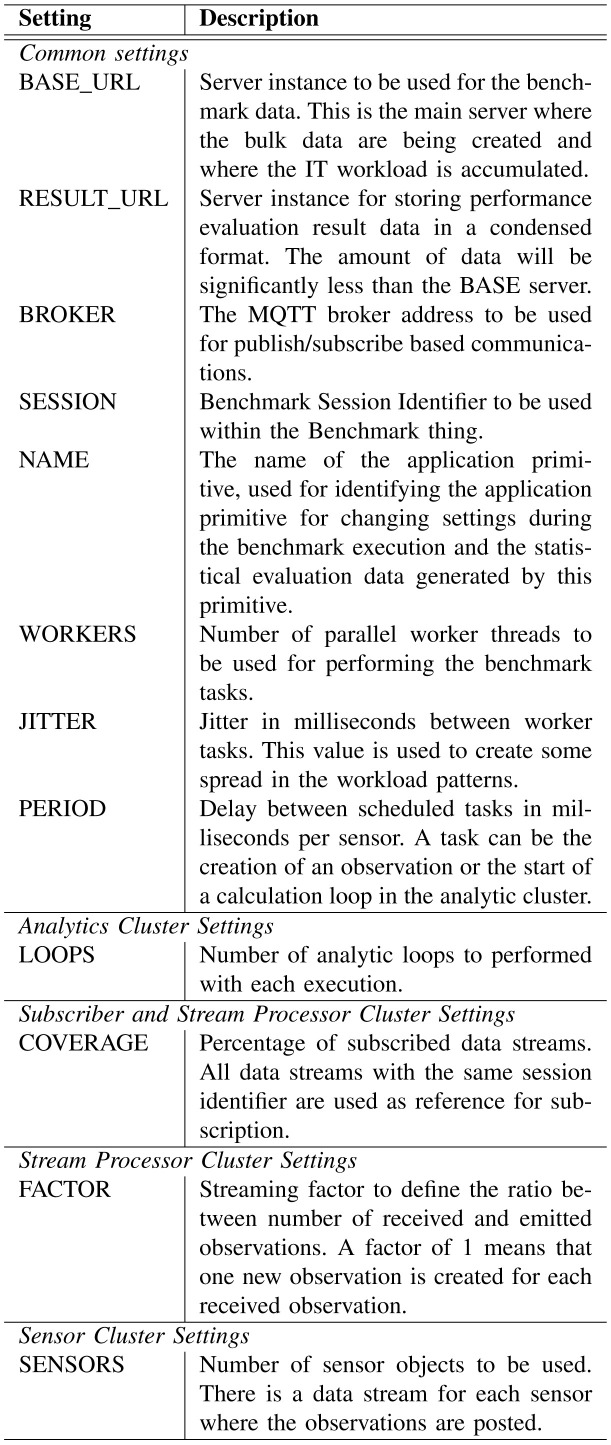
Application Primitive Settings.

The application primitives are configurable in several ways. The available settings are explained in Figure 3. Most settings are commonly used for each application primitive, some are specific. The settings can be given as environment parameters to the start process, or they can be modified during the execution (as explained later in section II-F).

### D. Operation

As the IoT Benchmark is implemented in Java without any specific requirements on the underlying hardware, it can be executed on virtually any operation system capable of running Java code. Also the performance requirements are only depending on the load configuration, so the application may also be used on low end hardware, like a *Raspberry Pi*.

However, the most typical environment for the application is a data center infrastructure, as it would be the case for many operational IoT applications. That is the reason why the build scripts are containing containerized versions for the software and also docker compose file ^12^ examples for the deployment of typical IoT application, as described on section III.

The IoT Benchmark contains a *Controller* application, which may be either used by an operator interactively as a command line interface, or it may be included in a composition as a scripted execution controller. An example for the scripting is shown in section II-G below. It is recommended to use the scripted controller in order to generate well defined and repeatable load pattern. The interactive command line mode is recommended for configuration testing and fine tuning.

### E. Benchmark composition

With the given application primitives it is now possible to compose specific IoT application patterns. Following the reference architecture patterns an IoT application may be composed of several IoT devices generating data, such as the example of the Microsoft IoT reference architecture ( Figure 1) shown above. These data sources can be emulated by one or more instances of the Sensor Cluster application primitives. Each sensor cluster produces sensor observations for a number of sensor objects with a rate as given by the settings shown in Figure 3.

Other elements of the IoT application may simply be added into a composition of application primitives. Each application primitive is designed to plug-in with other application primitives within the same session. For example, a Subscriber Cluster subscribes to sensor objects created by the Sensor Cluster, as defined by the configured *COVERAGE*. Likewise, the Stream Processor subscribes to the sensor objects, also based on the given coverage. Analytic Clusters implement business logic behavior by executing data selections from the data generated by the sensors of stream processors, combined with some compute intensive calculations to use some CPU power.

A composition of application primitives sharing the same SESSION identification, are considered to be one IoT benchmark application. It is possible to have multiple sessions at the same time, using the same database. However, this database will have to serve the accumulated IT load of all attached application primitives.

### F. Communication configuration

**Figure 4.**
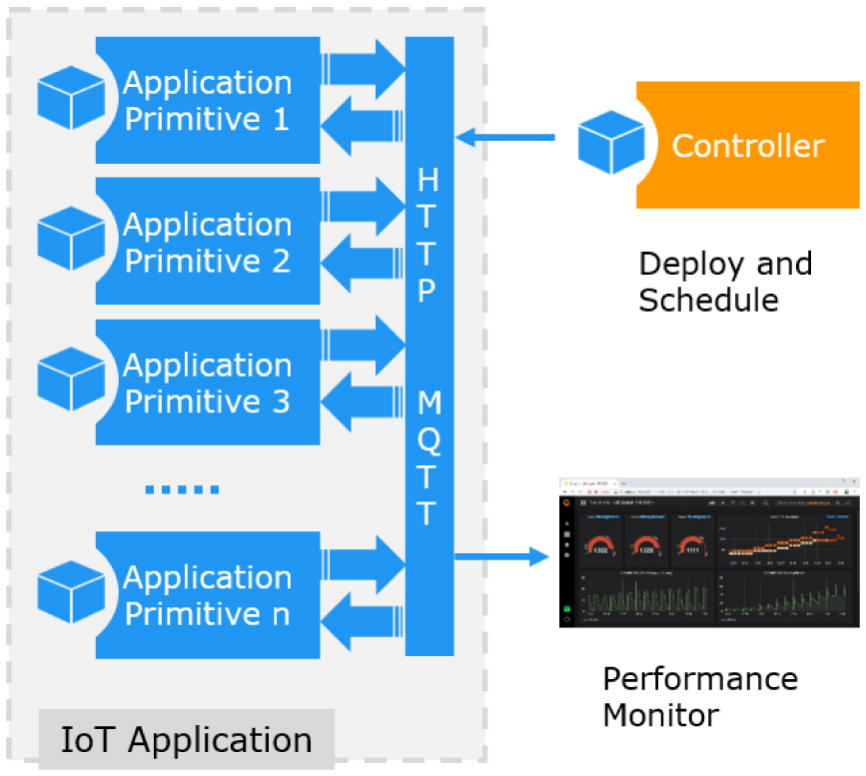
IoT Application Composition.

The application primitives are synchronized via the OGC SensorThings API ^13^ standard. This API is based on a data model using *Sensors* to create *Observations* to communicate events related to *Things*. The communication uses read/write and publish/subscribe communication patterns, based on a REST and MQTT protocol. Figure 4 shows the communication links between the application primitives.

The benchmark elements use the standard ^13^ for receiving the benchmark load specification, to synchronize with each other, and to generate the benchmark communication load. The load specification is communicated via a *Benchmark::Thing* with a set of session properties. The Controller publishes these properties and all other benchmark components are subscribed to. The benchmark load is created by temporarily instantiated *Benchmark::Sensor* objects, which are published, subscribed to, or filtered according to the application primitives.

Most data created during the IoT application execution are only temporary and will be removed after the application is terminated. Only summary performance data are published to a persistent database for monitoring and performance evaluation.

### G. Load schedule script

The Controller may also be used in a scripted mode, where the application does not offer a command line interface, but executes scripted statements. The script contains two elements, defined with a json formatted file. The first element defines the initialization parameters for each workload component. The second element defines a sequence of execution steps, where each step has a duration and a list of parameter changes to be used for that step. If no changes are given for a certain component in a certain step, the values from earlier steps, or the initialization settings of the built-in default values are used.

The configuration settings used for the initialization and for the execution phases, are structured as a properties list. Each element in the list has a name, which stands for the name of the load component, and a (key, value) list for the actual settings.

An example script is shown below:


{
    "initialize":
    {
         "S1": {
             "PERIOD": 1000000,
             "SENSORS": 500,
             "JITTER": 100000,
             "WORKERS": 10
         },
         "L": {
             "COVERAGE": 2
         },
         "A": {
             "PERIOD": 60000,
             "JITTER": 6000,
             "WORKERS": 10,
             "ANALYTICS": 0
         }
    },
    "sequence":
    [
         {
             "seq": 600,
             "info": "Hour 0600-0700",
             "duration": 10000,

             "S1": { "PERIOD": 600000},
             "L": { "COVERAGE": 2 },
             "A": { "PERIOD": 100000 }
         },
         {
             "seq": 700,
             "info": "Hour 0700-0800",
             "duration": 10000,

             "S1": { "PERIOD": 500000 },
             "L": { "COVERAGE": 2 },
             "A": { "PERIOD": 100000 }
         },
         ...
    ]
}


Listing 1. Application load configuration in JSON

This script provides initial parameter settings for three components and defines an execution script with two steps. Each execution step runs for 10000 ms, and provides changed parameter settings for some components.

### H. Availability

The IoT Workload Emulation software has been developed in a European funded project (see section VI) as Open Source software. It is available on a public repository in Github, see 14.

## III. IT Workload Definition

The IoT benchmark is designed to emulate any IoT application that fits into the architectural blueprint shown in II-B. Our approach to define the IT workload for a specific IoT application is to sketch a user story and break this down into smaller blocks until the application primitives are reached. The sections below explain this approach and show how configuration is done to create an emulation for a specific application.

### A. User story breakdown

The load on a computer system (CPU, storage, interfaces) is defined by the applications which run on it. Hence, low-level hardware-oriented load patterns are not in general a true reflection of the load generated by an application, cf. e.g. 1. A decompositional approach to defining application specific benchmark workloads is adopted here (similar to that in 8). This involves decomposing the application into functional layers as explained below. In fact, the approach here will start with User Stories of an Application Domain at the highest level, map (decompose) these to Use Cases, which in turn are mapped to Application Primitives. The Application Primitives may be organized in a layer hierarchy.

**Figure 5.**
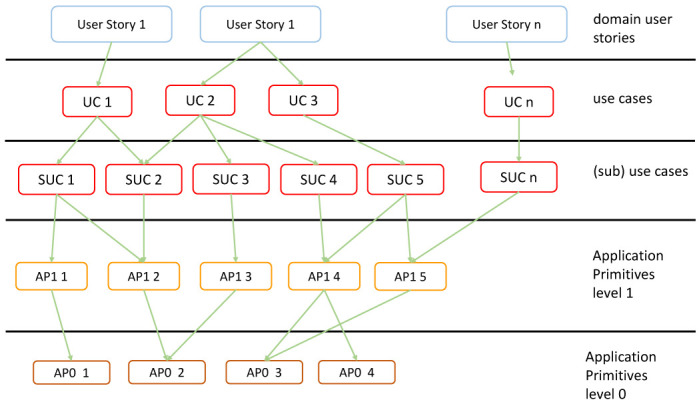
Application Domain Verticals.

The following key concepts will be used to define the high-level loads in the so-called load pattern characterization architecture (see Figure 5). 

- User Story: Describes in a few sentences in the everyday or business language of an actor; a situation that captures what a user does or needs to do as part of his or her job function. A user story captures the ‘who’, ‘what’ and ‘why’ of an activity in a simple, concise way. It is domain specific. A user story is sometimes referred to as an application scenario. It describes a situation and its context.
- Use Case: Describes functional aspects, actors and work-flows to be executed by the actors. An actor tells a user story, which motivates a use case to be performed by an actor. The use cases may be refined with sub-use cases. The use cases are mapped to Application Primitives (normally software modules) that are required to realize the respective use case.
- The Application Primitives are hierarchically grouped in layers of application primitives as shown with two levels of primitives. In general, there may be more levels. Primitives of a given level call primitives of the next lower level. The lowest level may be hardware-oriented operations such as the basic numeric operations or numeric routines such as matrix multiplication (e.g. on a GPU).

With the technique of application profiling, the idea is to find a load pattern that yields a close approximation to the real application load as measured close to the hardware and operating system. This is achieved by analyzing the measurement traces of an application.

### B. User Story Smart City example

In order to illustrate the approach of application profiling and mapping to application primitives, some examples from the Smart City domain are shown. Without aiming for completeness, the breakdown from the user story to use cases and then further to application primitives with illustrative configuration settings is listed below:

- **User Story Smart City** ^15^: A Smart city is an urban area that uses different types of electronic IoT sensors to collect data and then use insights gained from that data to manage assets, resources and services efficiently. This includes data collected from citizens, devices, and assets that is processed and analyzed to monitor and manage traffic and transportation systems, power plants, water supply networks, waste management, crime detection, information systems, schools, libraries, hospitals, and other community services.
  – **Use Case: Traffic Management**, to monitor individual traffic on public roads. Flow control based on light-signal installations and construction site regulation.
    * **Sub Use Case: Light-signal Control**, with 500 light-signal installations providing data updates every 100 ms and observed on a common data dashboard.
      • Application Primitive: SensorCluster (name=TrafficLights, sensor=500, period=100ms, jitter=5ms)
      • Application Primitive: SubscriberCluster (name= FlowControl, coverage=90%)
    * **Sub Use Case: Construction barrier**, with an average number of 20 road barriers for traffic control, providing monitoring data updates every minute. Status of all construction barriers is evaluated every minute.
      • Application Primitive: SensorCluster (name=RoadBarriers, sensor=120, period=60s, jitter=100ms)
      • Application Primitive: AnalyticsCluster (name=TrafficApp, loops=3000 M, period=60s)
    * Sub Use Case: Bicycle Monitoring, . . .
    * Sub Use Case: E-Bike Rental, . . .
    * Sub Use Case: Parking, . . .
  – Use Case: Harbor Port Management, . . .
  – Use Case: Environmental Monitoring, . . .

**Figure 6.**
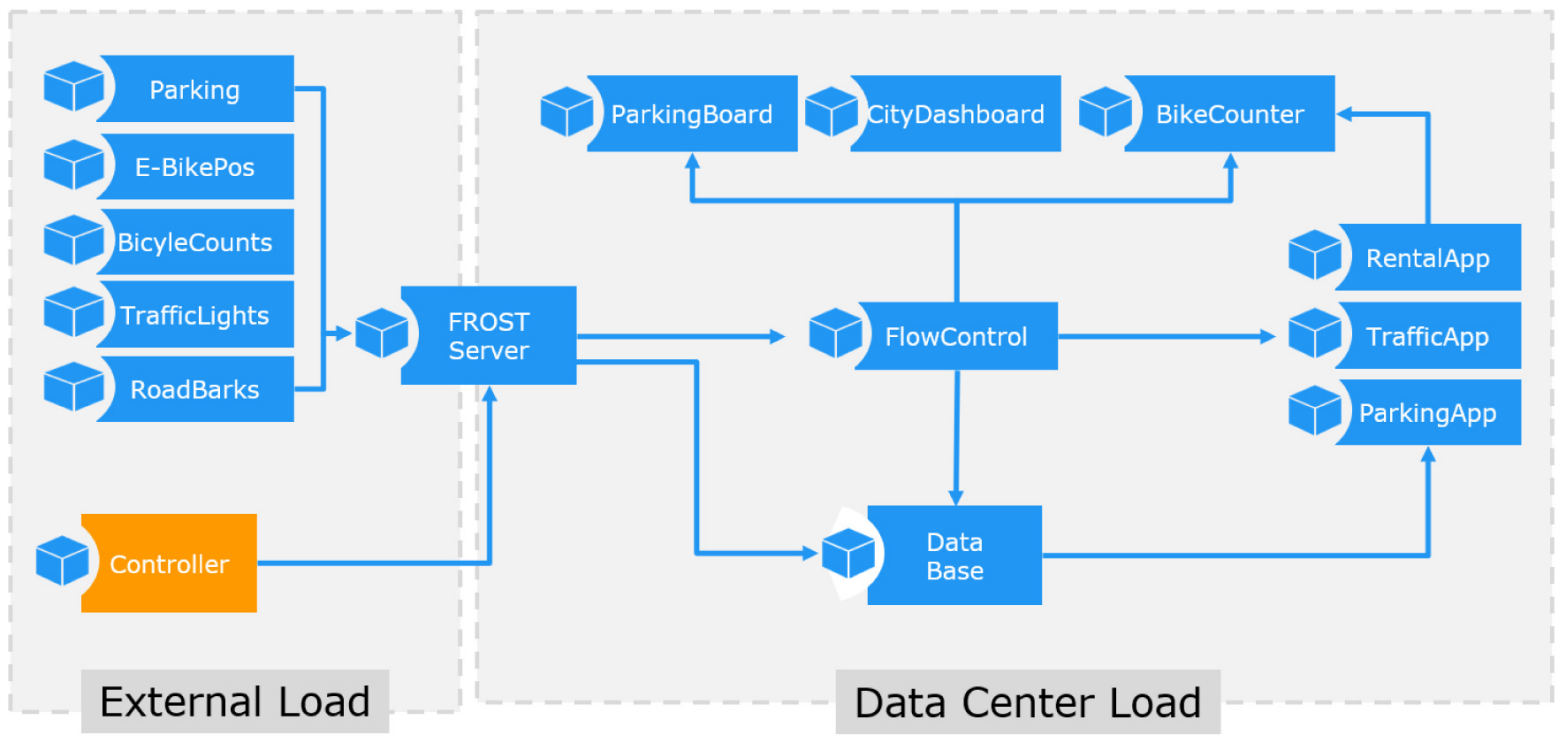
Application Primitives for Sub Use Case Traffic Management.

The IT workload is generated by the combination of all application primitives within the use cases. In Figure 6 the application primitives for the sub-use case *Traffic Management* are shown.

### C. Benchmark load

With the example given in III-B the communication load is defined as the sum of all included application primitives. For the use case traffic management example, with its two sub use cases, this leads to the following numbers:

- **Sub Use Case: Light-signal Control:** The first sub use case consists of two application primitives. A SensorCluster with the name *TrafficLights*, emulating 500 sensors, each sensor using a period of *100ms* and a jitter of *5ms* between observations. The jitter is only used to inject some noise into the distribution. The emitted load is calculated by the ratio of the number of sensors and the period of observations. The load is measured by the value *o [traffic light observations (obs) per second]*.

The second application primitive in this sub use case is the SubscriberCluster with the name *FlowControl*, and a coverage *90%* of all sensors. This load is measured by the value *s [subscriptions (sub) received per second]*.

$$O=\frac{sensors}{period}=\frac{500}{100ms}=5000[obs/sec]\;(1)$$

$$s=\frac{O*coverage}{100}=\frac{5000*90}{100}=4500[sub/sec]\;(2)$$

- **Sub Use Case: Construction barrier:** The second sub use case emulates a construction barrier scenario, also with two application primitives. A SensorCluster with the name *RoadBarriers*, emulating *120* sensors, each using a period of *60s* with a jitter of *100ms*. The sensor load is measured by the value *r [road barrier observations (obs) per second]*.

The second Application Primitive in this sub use case is a AnalyticsCluster with the name *TrafficApp*, performing *3000 M* analytic loops, with a period of *60* between each cycle. This load contribution is measured by *a [traffic evaluation (evl) loops per second]*.

$$r=\frac{sensors}{period}=\frac{120}{60s}=2[obj/sec]\;(3)$$

$$a=\frac{analytics}{period}=\frac{3000}{60s}=50[evl/sec]\;(4)$$

The overall load of a user story such as the *Smart City* is defined to be the sum of all application primitives in the included use cases. The result is the emulation of a single IoT application, as shown in Figure 6. 

## IV. The Benchmark Process

The benchmarks were trialed in practice as part of the BodenTypeDC project. Within the EU project, a real data center was built at Boden in north Sweden. The data center has four coolers with fresh air in front of two rows of servers. Each row consists of two triple racks, totaling 480 servers (40 servers in each sub rack). The software virtualization is done by Docker, Kubernetes and a workload scheduler implemented by RISE on top.

**Figure 7.**
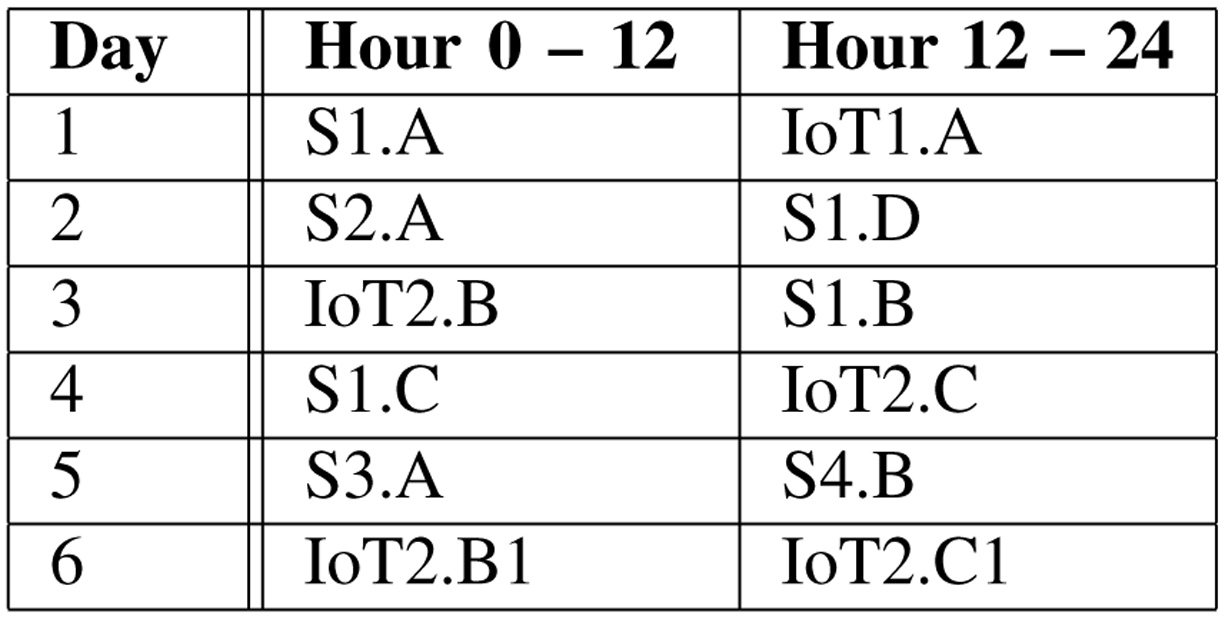
Measurement Campaign.

It was a great advantage to have a real test bed available to perform real measurements and to observe the influence of the climate variance during the year. Therefore, a measurement campaign was set up which was executed every three weeks and ran five days. The measurement campaign (see Figure 7) consists of various synthetic ( **S**) and application oriented ( **IoT**) workloads. The following number distinguishes different workloads and the capital letter stands for a distribution strategy. So IoT2.B is the second application oriented workload distributed on a B strategy. IoT1 stands for the smart city workload and IoT2 for the predictive maintenance workload.

Each workload runs 12 hours to have the opportunity to emulate the increase of the data during the day, to perform periodical analytic processes and emulate specific situations like rush hours or the end of a work shift.

Whereas the synthetic workloads are distributed with fixed parameters on the servers (e.g. 40% CPU utilization on each server for 2 hours, then additional 20% CPU utilization to the servers of racks 7 and 8), the application oriented workloads emulates real workload.

Each application consists of 14 connected and communicating tasks (described in sections II and III-B), including sensor data generating tasks, a database task and various analytic tasks. Each can be distributed on its own and generates communication traffic. The tests are so designed that all actions are performed within the data center, which means that also the sensor data are generated internally. Various applications are started in parallel to achieve a realistic rate of utilization on the data center. 120 applications started in one IoT workload generate a CPU utilization of about 35%. 

**Figure 8.**
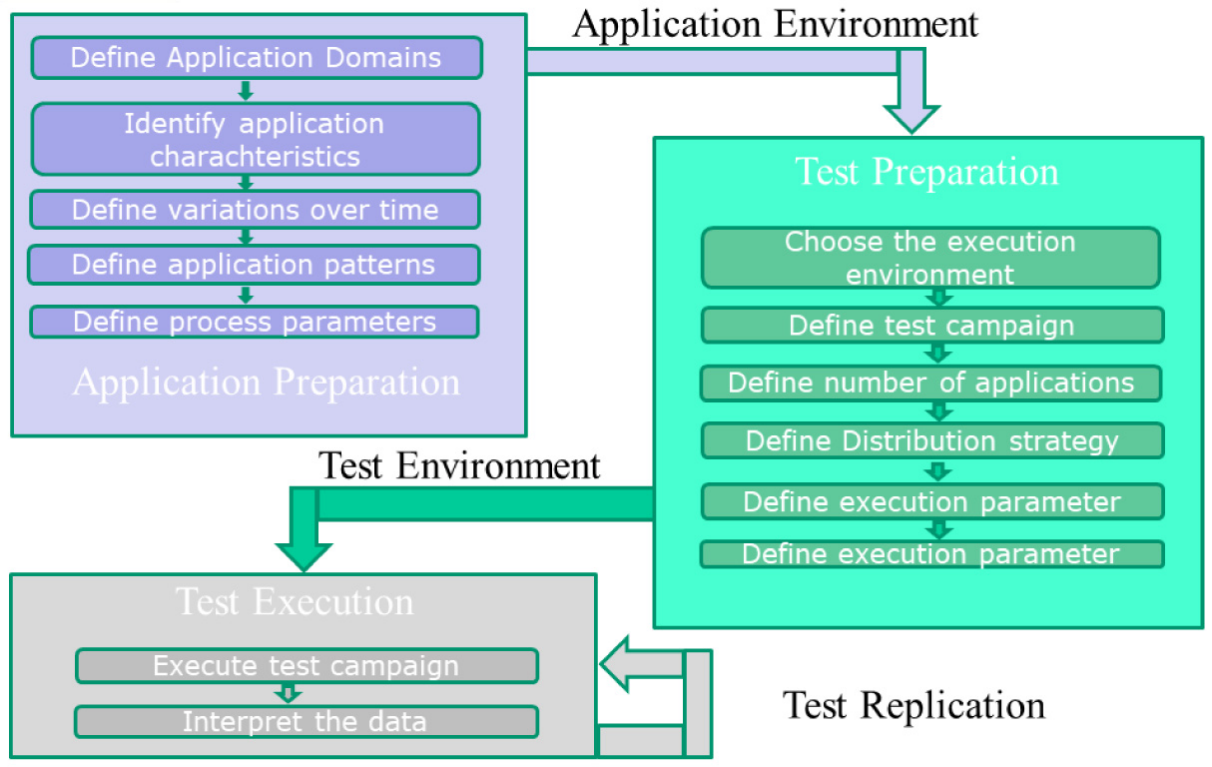
Benchmark Process.

The IoT benchmark process runs in three phases and within each phase in several steps (see Figure 8). The phases are *application preparation*, *test preparation* and *test execution*. 

**Application preparation:**

1) ***Define the application domains***

Select Smart Cities ^15^ or Predictive Maintenance ^16^

2) ***Identify the characteristics of the application areas.***

The application domain consists of sensors, a database, subscriber and analytic processes.

3) ***Decide if the data and process volume may differ during the test period.***

In the Smart City application domain the volume will change: the short morning rush hour, the midday rush hour, which will be a bit longer and the evening rush hour which lasted the longest.
In the Predictive Maintenance application domain the frequency of data and processing is production dependent. After a time period or the work shift it may increase.

4) ***Define a pattern for an application.***

In our case the application consists of 14 interconnected processes including a database.

5) ***Define the parameters for the process and vary them over the testing period.***

**Test preparation:**

1) ***Choose the execution environment.***

In this case BTDC One with 480 servers.

2) ***Define the test campaign.***

3) ***Define the number of applications which will be performed in the execution environment.***

Execute 120 applications here, but in two special workloads named IOT2.B1 and IOT2.C1 there are two time slots (each lasting 2 hours) where 120 additional applications are executed.

4) ***Choose the distribution strategies for the selected number of applications on the execution environment.***

Different distribution strategies (A, B, C, B1 and C1) are performed.

5) ***Define execution parameters such as cooling strategy or operating temperature interval.***

**Test execution:**

1) ***Execute***

2) ***Measure the execution parameters such as temperature, power usage, water usage, power usage effectiveness (PUE), pressure, fan speed etc.***

3) ***Interpret all the data.***

4) ***Change the environment and rerun the tests.***

## V. Results

The following figures show first impressions of the test results. Each figure includes three diagrams, the top diagram shows the CPU or the memory utilization, the middle the fan speed and the bottom the power usage diagram. Each diagram includes again various information. The legend of the diagram can be found beneath each diagram. The time period of the diagram can be seen in the headline of the figure on the right side.

The CPU (or the memory) utilization diagram shows four curves, they stand for the average of the CPU utilization in the four racks. Each rack is documented by a separate color. The utilization may differ depending on the distribution strategy and the load on the servers. The fan speed diagram shows the actual fan speed of two servers in each rack. The power usage diagram shows the power used and the PUE. It includes the power of the facility, the power of the IT and the total power, which belong to the left scale. The PUE, which is also included, uses the right scale.

**Figure 9.**
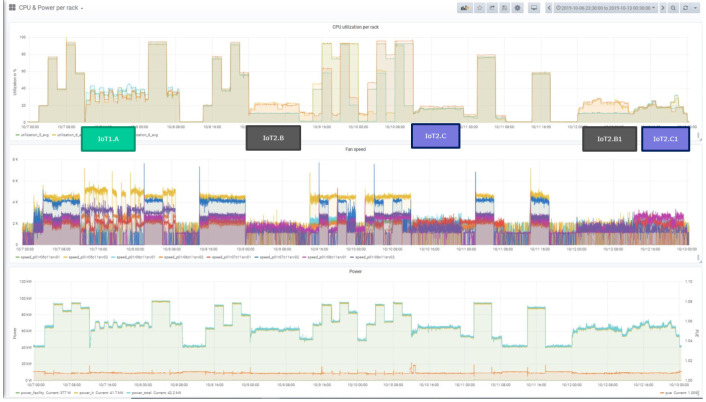
CPU utilisation and power consumption of the IoT workloads during a measurement campaign (Test Campaign 11).

Figure 9 shows the CPU utilization, the fan speed, the power usage and the PUE for the eleventh test campaign (TC 3) which took four days. It is obvious that the application-oriented workloads (IoT1.A, IoT2.B and IoT2.C) are much more dynamic than the synthetic workloads performed during the other time periods ^17^. The fan speed and the power consumption are significant indicators. It can also be seen that one IoT workload differs from the others. The workload is also shown for the different racks.

**Figure 10.**
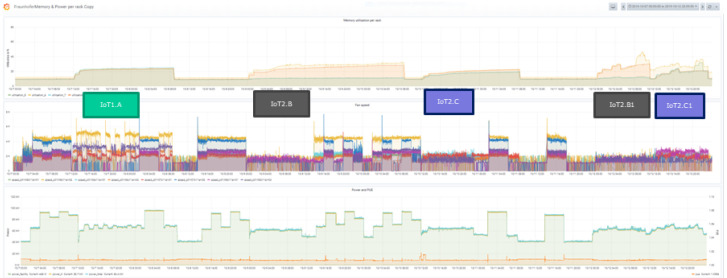
Memory utilisation and power consumption of the IoT workloads during a measurement campaign (Test Campaign 11).

Figure 10 shows the memory utilization and power consumption of the eleventh test campaign. The IoT workloads are marked and the different behavior compared to the synthetic workloads can be seen. It can also be seen that the IoT workloads have an increasing memory consumption over time.

**Figure 11.**
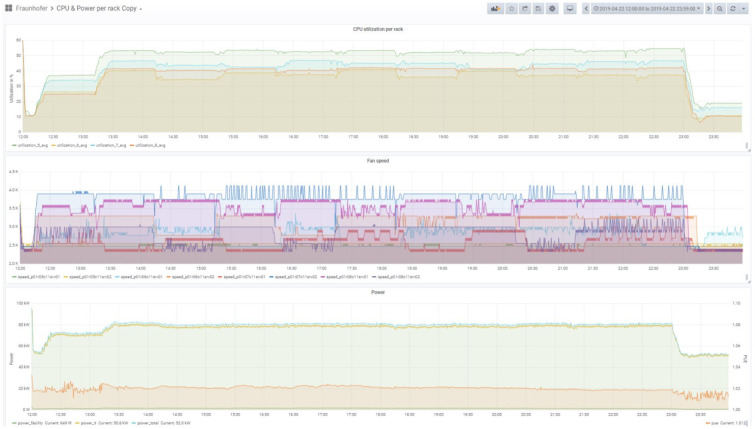
CPU utilisation and power consumption of the IoT workloads 1A – Smart City (Test Campaign 3).

Figure 11 shows the CPU utilization and power consumption of the smart city workload in the third test campaign. A look at the start phase shows that first the synthetic background load is started and then in various steps the IoT loads. It takes some time until all processes are started and the communication processes are established. The four racks (represented with different colors) are visible with their variable values. The PUE reacts to the changed load, but stays in a small bandwidth.

## VI. Conclusion

The initial motivation for the IoT benchmark was the requirement within the BTDC project to have a realistic load generator to represent a typical data center IT workload. Several market development predictions indicate that the IoT application domain, with all its sub-domains like Industrial IoT, healthcare, smart cities, environmental sciences, and many others, will become one of the biggest IT workload generators for the data center industry. That is the market-driven reason to choose the IoT domain as a benchmark to represent typical IT-workloads.

The second reason was the architectural and structural uniformity of the IoT applications. Even though an impressive number of technical IoT solutions have been created, interestingly enough, the architectural approaches show many commonalities among their structural features. The reference architectures of various standards developing organizations are very similar as to how data is created by *Things*, and communicated via the *Internet*, and how the data is being processed, mostly somewhere in the *Cloud*. This allows to abstract from many IoT applications to a relatively small number of application primitives (see section II-C) as building blocks resembling the actual behavior of a real application. These application primitives have been designed as highly configurable and reproducible building blocks to create workload experiments, such as the testing of the cooling efficiency of a data center under “realistic” workload conditions.

The IoT Workload Emulation has been used to emulate various types of IoT applications to create an adequate IT workload for an industrial grade data center. The software is available as open source, see 14. 

A further use case for the IoT workload emulation is the modeling of specific application loads, in order to test deployment concepts for future IoT applications, or expected load conditions. As the workload emulation software is highly configurable and easy to deploy, it is possible to create emulations of many different types of IoT applications in order to test and predict requirements on server and communication infrastructure. This is especially useful for applications where it is not easy to change the actual load, because it might depend on external events which are difficult to change, such as the number of users or connected IoT devices.

## Data availability

Fordatis: Open Research Data Pilot - BTDC. http://dx.doi.org/10.24406/fordatis/87 ^17^. 

 Data are available under the terms of the Creative Commons Attribution-NoDerivatives 4.0 International license (CC BY ND 4.0). 

## Software availability

Source code is available from: https://github.com/FraunhoferIOSB/IoT-Benchmark.

Archived source code at time of publication: https://doi.org/10.5281/zenodo.4435095 ^9^.

License: GNU Lesser General Public License version 3. 
