# Supplementary material for: IoT Workload Emulation for Data Centers
Source: Open Res Eur. 2021 Mar 24;1:12. [Version 1] doi: 10.12688/openreseurope.13079.1 (PMC10445864; doi:10.12688/openreseurope.13079.1)
Supplement: Peer review history

# Peer review: Reviewer response for version 1

**DOI:** 10.21956/openreseurope.14149.r27397

**Published:** 2022-02-28

**Authors:** Salman Zubair Toor

**Affiliations:** 1 Department of Information Technology, Division of Scientific Computing, Uppsala University, Uppsala, Sweden

The article addresses an important challenge of emulating different IoT workloads. It is a serious effort that provides a basis for future contributions in the field by offering scientific insights, an open source framework and a related dataset. Overall the article is well articulated and easy to read. I think the article requires minor changes. 

 Following are my comments:

1. The introduction section doesn’t highlight why workload emulators are really required? The only motivation provided in the first half is “The motivation for the development of an IoT Workload Emulation originated in the H2020 BodenTypeDC project.” The authors need to highlight the significance from the view of what challenges workload emulators to address and also the ongoing efforts.  (Related to question 1 - Is the rationale for developing the new software tool clearly explained?)
2. The idea of the third approach (algorithmically defined) for the emulator design is interesting however it is important to highlight what use cases it will cover that other approaches (empirical and mathematical) cannot address? (Related to question 2 - Is the description of the software tool technically sound?)
3. It is always good to explain the domain-specific abbreviations, such as MQTT.
4. Section II-H contains exactly the same information as in the first paragraph of section II-C. Some explanation will be required to understand the difference between these two paragraphs.
5.  I suggest to further extend section II-A with more state-of-the-art approaches in the field. Also, highlight the current pressing challenges in the area.
6.  Authors highlighted easy to handle, reproducible, scalable and configurable as the features of an emulator framework. From the presented use cases, some features in the proposed framework are visible such as reproducible and configurable. However, it is unclear how the proposed framework offers other mentioned features, easy-to-use and scalable.
7. The labels in Figures 9 to 11 are very hard to read. 

Is the rationale for developing the new software tool clearly explained?

Partly

Is the description of the software tool technically sound?

Partly

Are sufficient details of the code, methods and analysis (if applicable) provided to allow replication of the software development and its use by others?

Yes

Is sufficient information provided to allow interpretation of the expected output datasets and any results generated using the tool?

Partly

Reviewer Expertise:

Distributed Computing Infrastructures, Applied Machine Learning, Clouds and BigData.

I confirm that I have read this submission and believe that I have an appropriate level of expertise to confirm that it is of an acceptable scientific standard.

# Peer review: Reviewer response for version 1

**DOI:** 10.21956/openreseurope.14149.r26651

**Published:** 2022-02-21

**Authors:** Volker Coors

**Affiliations:** 1 Faculty of Geomatics, Computer Science and Mathematics, Hochschule für Technik Stuttgart, Stuttgart, Germany

The authors of the paper "IoT Workload Emulation for Data Centers“ propose a new method to emulate the workload of a data center by typical IoT applications. The scope of the emulator is clearly defined and excludes the IoT devices (“Things”) itself, which is reasonable. The emulator was evaluated with measured workload of an existing data center at Boden in Sweden. The developed software is open source and can be configured and used in other use cases as well.

 The paper is technically sound. The components and APIs used are state of the art and representative. For instance, the SensorThings API is widely used in Smart City applications.

 Major issue:

 Figures 9, 10, 11 are hard to read. Do they display the measured workload or the emulated workload as well? Do the figures show a comparison of measured and emulated workload? It is difficult to see as I can’t read the labels. The resolution of the figures need to be improved.

 Minor issue:

- The list of sub use cases looks like a draft. I would recommend to remove the bullet point list and add a sentence: “addition sub Use Cases such as bicycle Monitoring, E-Bike Rental, etc. can be defined the same way.”
- Some sentences are written in “german-english”: “Test tests are so designed […]”

Is the rationale for developing the new software tool clearly explained?

Yes

Is the description of the software tool technically sound?

Yes

Are sufficient details of the code, methods and analysis (if applicable) provided to allow replication of the software development and its use by others?

Yes

Is sufficient information provided to allow interpretation of the expected output datasets and any results generated using the tool?

Partly

Reviewer Expertise:

computer science, geoinformatics

I confirm that I have read this submission and believe that I have an appropriate level of expertise to confirm that it is of an acceptable scientific standard, however I have significant reservations, as outlined above.

# Peer review: Reviewer response for version 1

**DOI:** 10.21956/openreseurope.14149.r26941

**Published:** 2021-06-07

**Authors:** Karim Djemame

**Affiliations:** 1 School of Computing, University of Leeds, Leeds, UK

The motivation for this research on Internet of Things (IoT) benchmarking is the requirement to have a realistic load generator to represent a typical data centre IT workload. The paper therefore introduces an approach to decompose IoT applications into application primitives, and use them to emulate a workload as it would be created by the modelled application. To define the IT workload for a specific IoT application, a user story is sketched and broke down into small blocks until the application primitives are reached. An evaluation is performed to trial the benchmarks in practice as part of the BodenTypeDC project where a “real” data center is considered to test cooling approaches under realistic work load conditions.

 The article is timely and technically sound. Some comments are included in order to improve further its quality.

1. Include a stronger motivation paragraph in the introduction: what is the problem? Why is it important to address it? What are the limitations of the current solutions (if any)?
2. Include a section on related work on emulation/simulation of IoT, e.g. MAMMOTH a large scale emulation platform for IoT.
3. Avoid referencing sections in the paragraphs 1 and 4 in the introduction. Rather, include a paragraph at the end of the section describing the structure of the article.
4. Use the taxonomy of IoT applications to derive useful information with regard to their workload. This information can also be the driver for the benchmark.
5. In section II-C Implementation, it is preferable to first introduce the components, roles and interaction and then the implementation details (Java, Maven etc).
6. The quality of figures 9-11 can be improved.
7. Section 4 – Benchmark Process. It is unclear whether the synthetic workloads are actually used in the experiments.
8. In Figures 9 and 10, explain the differences between fan speed and power consumption. Are they identical in both figures?
9. It will be good to include a discussion on the scalability aspects: 1) what if the IoT workload increases by 10, 100? And is allocated on the same number of servers in the data centre? What if the number of servers is increased? Would the benchmark be able to cope with such scenarios? Would the same results as those in section 5 be expected? Same correlation between the metrics?

Is the rationale for developing the new software tool clearly explained?

Yes

Is the description of the software tool technically sound?

Yes

Are sufficient details of the code, methods and analysis (if applicable) provided to allow replication of the software development and its use by others?

Yes

Is sufficient information provided to allow interpretation of the expected output datasets and any results generated using the tool?

Yes

Reviewer Expertise:

Distributed systems

I confirm that I have read this submission and believe that I have an appropriate level of expertise to confirm that it is of an acceptable scientific standard.
